# Accuracy of 3-Dimensionally Printed Full-Arch Dental Models: A Systematic Review

**DOI:** 10.3390/jcm9103357

**Published:** 2020-10-20

**Authors:** Yasaman Etemad-Shahidi, Omel Baneen Qallandar, Jessica Evenden, Frank Alifui-Segbaya, Khaled Elsayed Ahmed

**Affiliations:** School of Dentistry and Oral Health, Griffith University, Griffith Health Centre (G40), Office: 7.59, Brisbane, QLD 4215, Australia; yasaman.etemadshahidi@griffithuni.edu.au (Y.E.-S.); omelbaneen.qallandar@griffithuni.edu.au (O.B.Q.); jessica.evenden@griffithuni.edu.au (J.E.); f.alifui-segbaya@griffith.edu.au (F.A.-S.)

**Keywords:** 3-dimensional printing, additive manufacturing, dental models, accuracy, systematic review, full-arch

## Abstract

The use of additive manufacturing in dentistry has exponentially increased with dental model construction being the most common use of the technology. Henceforth, identifying the accuracy of additively manufactured dental models is critical. The objective of this study was to systematically review the literature and evaluate the accuracy of full-arch dental models manufactured using different 3D printing technologies. Seven databases were searched, and 2209 articles initially identified of which twenty-eight studies fulfilling the inclusion criteria were analysed. A meta-analysis was not possible due to unclear reporting and heterogeneity of studies. Stereolithography (SLA) was the most investigated technology, followed by digital light processing (DLP). Accuracy of 3D printed models varied widely between <100 to >500 μm with the majority of models deemed of clinically acceptable accuracy. The smallest (3.3 μm) and largest (579 μm) mean errors were produced by SLA printers. For DLP, majority of investigated printers (*n* = 6/8) produced models with <100 μm accuracy. Manufacturing parameters, including layer thickness, base design, postprocessing and storage, significantly influenced the model’s accuracy. Majority of studies supported the use of 3D printed dental models. Nonetheless, models deemed clinically acceptable for orthodontic purposes may not necessarily be acceptable for the prosthodontic workflow or applications requiring high accuracy.

## 1. Introduction

Three-dimensional (3D) printing is an additive manufacturing (AM) process that allows conversion of digital models into physical ones through a layer-by-layer deposition printing process. 3D printing has been adopted in dentistry at an increasing rate and construction of dental models is one of the main applications of this promising technology in prosthodontics, orthodontics, implantology and oral and maxillofacial surgery, amongst others [1]. An essential prerequisite of dental models is creating an accurate replication of teeth and the surrounding tissues to serve their intended purposes as diagnostic and restorative aids for assessment, treatment planning and fabrication of various dental appliances and prostheses. Currently, gypsum casts poured from conventional impressions (e.g., alginates silicones, poly-sulphurs, ethers) are considered the gold standard for constructing dental models [2]. However, these cast models suffer a number of limitations, including a need for expedited processing of impressions, depending on the impression material; storage space for resultant casts; the cost of human and laboratory resources involved in fabrication; poor structural durability; and a propensity to dimensional changes over time [3]. In contrast, 3D printed models could offer a more efficient workflow that can be manufactured on demand and are more resilient, less-labour intensive and potentially time-saving [4]. Nonetheless, 3D printed models also present a unique set of limitations. The accuracy of the resultant models depends on several factors that can introduce errors. This includes the data acquisition and image processing of the oral hard and soft tissues, and the myriad of parameters involved in the manufacturing and postprocessing processes [5]. Moreover, models acquired through vat polymerisation and material jetting are prone to shrinkage during the polymerisation stage as well as having stair-step surfaces due to the layering technique used in construction [6]. In addition, a recent study demonstrated that models exhibit dimensional changes postprocessing as they age with their dimensions reported to be significantly different after three-weeks of manufacturing [7].

At present, there is an array of printing technologies available utilising various techniques, with varying outputs and performances, and consequently confounding the issue of a standardised expectation of accuracy. The most commonly used techniques are stereolithography (SLA), digital light processing (DLP), material jetting (MJ) and fused filament fabrication (FFF). Other processes such as continuous liquid interface production (CLIP) and binder jetting (BJ) have also been utilised but are not as common [8]. The earliest and most widely adopted 3D printing technique is SLA, which utilises ultraviolet (UV) scanning laser to sequentially cure liquid photopolymer resin layers. Each layer is solidified in the x-y direction, and the build platform incrementally drops in the z-direction to be recoated by resin and cured [9]. The photopolymerisation of each new layer connects it to the prior layer resulting in models with good strength. DLP uses a conventional light source to polymerise photosensitive liquid resins. However, unlike SLA, each x-y layer is exposed to the light all at once using a selectively masked light source, resulting in shorter production time [10]. Both SLA and DLP are versatile techniques as they can be used with a wide variety of resin systems [11]. CLIP is an advanced form of DLP technology with the advantage of faster printing time. Additionally, this technique utilises a membrane, which allows oxygen permeation to inhibit radical polymerisation. MJ, similar to vat polymerisation techniques (SLA, DLP and CLIP) employs photopolymerisation. This technique allows for deposition of liquid photosensitive resin through multiple jet heads on a platform, which is then cured by UV light [12]. As opposed to SLA and DLP, this technique requires no post-curing. Unlike Vat polymerisation and MJ, which use photopolymer material, FFF relies on the melting of thermoplastic materials, extruded through a fine nozzle, to create objects through layering filaments [11]. BJ technology, on the other hand, utilises selectively deposited liquid bonding agents to fuse powdered material.

The International Organization for Standardization (ISO 5725-1:1994) identifies accuracy as a qualitative concept, with trueness and precision being its quantitative counterparts. Trueness is defined as the ‘closeness of agreement between the arithmetic mean of a large number of test results and the true or accepted value’. Precision is defined as the ‘closeness of agreement between test results’ [13]. There is currently no systematic review of data published on accuracy of dental models manufactured using 3D printing technologies; henceforth, this review aims to investigate the existing literature and evaluate the accuracy of 3D printed dental models using different 3D printing technologies and identify the printing parameters influencing their accuracy.

## 2. Materials and Methods

### 2.1. Review Question

The review search question was formulated using the PICO principle (Population, Intervention, Control, Outcome) [14], with dental models as the population cohort, 3D printing as the intervention and accuracy as the outcome. No control was defined. Hence, the formulated question was, “What is the accuracy of dental models manufactured using 3D printing technologies?” The protocol was registered on PROSPERO (registration number: CRD42020164099). The PRISMA guidelines were followed, where applicable [15].

### 2.2. Eligibility and Search Strategy

An electronic databases search was performed for PubMed, Cochrane Database, Web of Science, Scopus, EMBASE, LILACS, Scientific Electronic Library Online (SciELO) and the first ten pages of Google Scholar, using keywords and MeSH terms (Table 1). The Peer Review of Electronic Search Strategies (PRESS) guidelines were followed with an independent peer-reviewing the suitability of the search strategy [16]. Additionally, hand searching and cross-referencing was performed to identify additional studies. All study designs were included, whether prospective, retrospective, experimental in-vivo or in-vitro. The studies were limited to those published in English in the past 15 years (from 1 January 2005 to 13 March 2020). Abstracts from conferences, letters to the editor and studies that did not assess the accuracy of human dentate dental arches were excluded.

Initial screening of the titles and abstracts was independently performed by two investigators (O.Q. and J.E.). A list of the selected papers was compiled and compared, and any disagreements were discussed with a third investigator (K.A.) until a consensus was reached. Thereafter, the full text of the selected articles was reviewed to confirm the fulfilment of the inclusion criteria.

### 2.3. Data Extraction

Inclusion criteria and trial quality of included articles were assessed individually by two investigators (O.Q. and J.E.). The selected data were independently extracted and then cross-checked between the investigators and discrepancies were resolved by referring to a third investigator (K.A). Data collection, extraction and synthesis of the included studies was performed according to the following criteria:

- Sample size;
- model type;
- the 3D printing technology used;
- resolution (x,y) and layer thickness (z) used;
- materials and postprocessing protocol;
- accuracy of intraoral/lab scanner;
- accuracy assessment methodology;
- measurement of dimensional accuracy over time;
- presence of a study control;
- findings (accuracy); and
- limitations.

The authors of the included studies were not contacted to provide missing data not reported in their published studies.

### 2.4. Statistical Analysis and Risk of Bias (Quality) Assessment

A quality assessment of the methodology of the included studies was performed using the quality assessment of diagnostic accuracy-2 (QUADAS-2) [17] to assess their risk of bias and applicability concerns. Each domain was assessed and ranked as high risk, low risk or unclear.

## 3. Results

**Figure 1:**
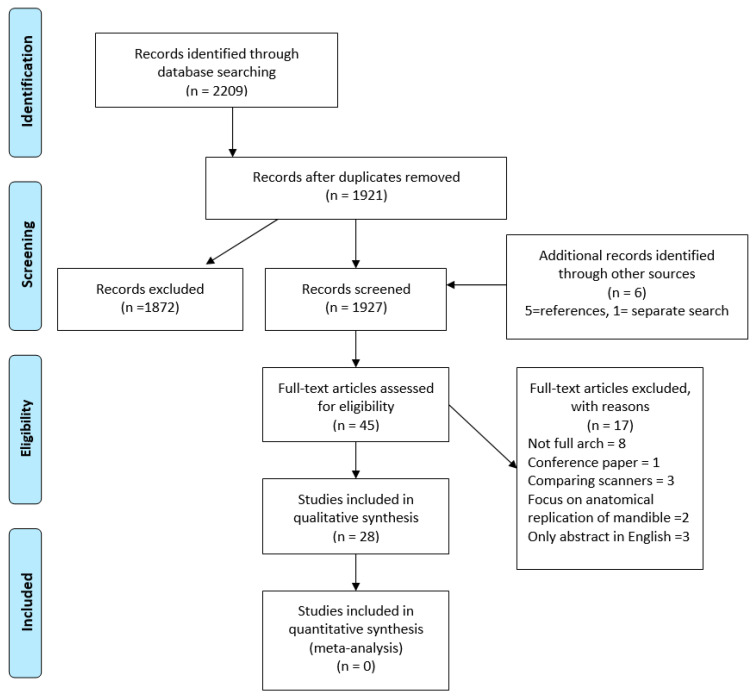
Flow chart for the selection of studies.

A total of 2209 studies were initially identified after the databases search (Figure 1). Screening of the titles and abstracts, and removing duplicates, resulted in 39 studies being selected. Six additional studies were identified through cross-referencing. Excluded studies either did not assess full-arch dental model [18,19,20,21,22,23,24,25,26,27] or were not published in English [28,29,30]. Three additional studies were later removed as they assessed and compared the accuracy of different intraoral scanners [5,31,32]. In addition, one study [33] was excluded as it was a published abstract. Finally, twenty-eight studies fulfilled the inclusion criteria and were further synthesised.

### 3.1. Study Characteristics

#### 3.1.1. Sample Size and Reference Models

For this study, the sample size was determined based on the number of single dental arches manufactured by each printer. The majority of the studies (*n* = 19/28) assessed models of both maxillary and mandibular arches, and the remainder used either the maxillary (*n* = 8) or mandibular (*n* = 1) arches. The sample size ranged between one and sixty 3D printed single arch models per printer (Table 2).

#### 3.1.2. Sample Details and Controls

The inclusion criteria for the studies that collected patient samples (digital or physical impressions or models) varied slightly with the majority (*n* = 14/25) being full arch dentate post-orthodontic models, including up to permanent first molars [36,37,38,39,42,44,46,47,48,49,52,55,56,57]. One of the studies [42] also used a model with a shortened dental arch. Another used an edentulous mandibular cast with four multi-unit abutments for implant prosthodontic rehabilitation [51].

Twenty-four studies included reference models as controls in their methodology design [6,7,34,36,37,38,39,40,41,42,43,44,45,46,47,48,49,50,51,52,53,55,56,57]. The controls included were a dental stone cast (*n* = 8), a digital STL image of a dental stone cast (*n* = 3), typodont digital STL image (*n* = 7), typodont (*n* = 1), prefabricated resin model digital STL image (*n* = 2), patient intraoral scan image (2) or a dry human skull (*n* = 1). In addition, there were four studies [4,35,47,54] that did not include a reference model as a control, rather compared various printing technologies against each other.

### 3.2. Additive Manufacturing

#### 3.2.1. D printing Technologies Assessed and Printing Parameters

An array of additive manufacturing systems were assessed in the included studies with several investigating more than one type of technology, printer brand or parameter settings (Table 2). The majority of studies investigated SLA (*n* = 20), MJ (*n* = 11), DLP (*n* = 9) and, to a lesser extent, FFF (*n* = 6), BJ (*n* = 2) and CLIP (*n* = 1). With regards to printing parameters, one study reported following the manufacturers’ recommendations [6], while others explicitly detailed the printing parameters used [4,19,36,37,38,42,43,45,46,47,49,50,51,52,55,56,57]. In contrast, the remainder of the studies did not provide clear details regarding the printing parameters used.

#### 3.2.2. Layer Thickness

The specified printing layer thickness (z-axis resolution) substantially varied amongst studies and ranged from 25–150 μm for SLA, 20–100 μm for DLP, 16–32 μm for MJ, 100–150 μm for FFF and 89–102 μm for BJ. The study using CLIP technology did not specify the layer thickness used [53]. Most studies did not specify the printing resolution in the x- and y-axes. However, in those that did, the x-y plane resolution ranged from 50–140 μm for SLA, 50–70 μm for DLP and 12.5–200 μm for FFF.

#### 3.2.3. Materials Used

The materials used by 3D printers are broadly classified based on their printing technologies. Vat polymerisation technologies (SLA, DLP and CLIP) used liquid photopolymers, including acrylates and epoxides, 3D material extrusion technology (FFF) used polylactic acid (PLA), or acrylonitrile butadiene styrene (ABS). MJ technology used photopolymers resins (acrylates) in liquid form and BJ technology used polylactic acid powder. Eleven studies did not specify the material used for the corresponding technology [19,35,36,40,44,45,48,52,53,54,55]. Within the studies assessing stone models, four used Type IV dental stone [6,35,40,45], one used Type III dental stone [4] and one did not specify the stone type utilised [34].

#### 3.2.4. Base Designs and Filling Patterns

The three types of base designs used in the studies were horseshoe-shaped bases [4,34,36,37,38,39,41,43,49,50,51,55], regular American Board of Orthodontics (ABO) [35,38,44,46,47,49,52,54,57] and horseshoe-shaped with a transverse supporting bar [7,38,48,53]. Six studies did not specify their base design [6,19,40,42,45,56]. Filling patterns employed in these studies were predominantly solid; however, hollow shelled [53,55] and honeycomb [37] were also utilised.

#### 3.2.5. Postprocessing Protocol

The majority of studies did not specify the postprocessing protocol (*n* = 19/28) [6,19,34,35,36,40,41,44,45,46,47,48,50,51,52,54,55,56,57]. Nine studies reported their post-curing protocol for vat polymerisation techniques [4,7,37,38,39,42,43,49,53] which included cleaning the models with isopropyl alcohol [37,43,49,53], or ethanol [4] to remove uncured resin followed by curing with UV light. Three studies only used UV light to post-process SLA models [38,39,42]. One study placed the SLA models in an ultrasonic bath followed by using light with wavelengths of 280–580 nm for post-curing [7]. Two studies reported that MJ and FFF did not require post-curing [37,38], and one study rinsed the MJ printed models in a bath of caustic soda to clean them [42]. Additionally, three studies specified removing the support structures from the models [37,42,57].

### 3.3. Assessment Methodology

The assessment of the accuracy of 3D printed models was performed using either 3D deviation analyses or 2D linear measurements. For the 3D assessment, step-height measurements through iterative point-cloud surface-matching followed by 3D deviation assessment were performed. For 2D linear measurements, reference points were selected and measured either directly onto the physical model using digital callipers or indirectly on the model’s digital image using surveying software. The majority of studies relied on 3D assessment [4,6,7,37,39,40,42,43,45,48,51,53,57] followed by digital callipers [34,36,39,41,44,46,47,52,54,55,56] and surveying software measurements [19,35,38,50]. One study [49] used the ABO cast-radiograph evaluation tool.

#### 3.3.1. Surface Matching and 3D Deviation Analyses

The studies which performed 3D assessment used min/max nominal values ranging between ± 10 to ± 60 μm and min/max critical values of ± 100 to ± 500 μm. Before superimposition, the 3D-printed models were scanned and converted to standard tessellation language (STL) format. The scanners included desktop/laboratory scanners (*n* = 15) [4,6,7,19,35,37,38,40,42,43,45,48,51,53,57], intraoral scanners (*n* = 2) [36,41], and computerised tomography scanner (*n* = 1) [38]. Two studies did not specify the details of image acquisition [34,50]. While most studies did not specify the accuracy of the scanners nor mentioned calibrating the scanners before scan acquisition, the remaining studies reported a scanning accuracy <20 μm [4,6,19,40,45,46,57].

#### 3.3.2. Linear Measurements of Physical and Digital Models

Studies that utilised digital callipers with physical models or measuring software with digital models relied on various reference points to perform 2D linear measurements. The reported accuracy of all callipers was 10 μm, and the ABO tool was 100μm. The selected reference points relied on varying tooth measurements (crown height, mesiodistal width, buccolingual width and marginal ridge width), arch measurements (intercanine width, interpremolar width and intermolar width) and occlusion measurements (overjet, overbite, occlusal contact and interarch sagittal relationships). Most studies used both tooth and arch measurements (*n* = 10) [19,34,36,39,41,43,46,47,55,56], while one study only used tooth measurements [44] and three studies only used arch measurements [35,38,40]. Moreover, five studies used occlusion measurements in addition to the arch measurements [19,39,49,52,54].

#### 3.3.3. Time of Assessment

The time at which the 3D printed models were scanned or measured was reported by six studies [7,41,45,46,47,52]. Within those studies, five assessed the models after a week of printing [41,45,46,47,52] and one assessed the accuracy after one day, followed by weekly intervals for four consecutive weeks [7].

### 3.4. Outcomes Assessed

#### 3.4.1. Clinical Acceptability

The clinically acceptable error defined in the studies varied widely from <100 μm [51,53], < 200μm [6,45], <250 μm [43], <300 μm [44,48] and <500 μm [19,34,35,36,42,46,47,49,50,52,55,56,57]. One study [4] defined various acceptable ranges of error for different measurement points and seven studies did not define any clinically acceptable range [4,7,37,38,40,41,54]. From those, twelve assessed orthodontic models [19,36,39,42,43,44,47,49,52,55,56,57], five assessed fixed pros and implant models [6,34,35,51,53] and three did not specify [45,46,48].

#### 3.4.2. Trueness

**Figure 2:**
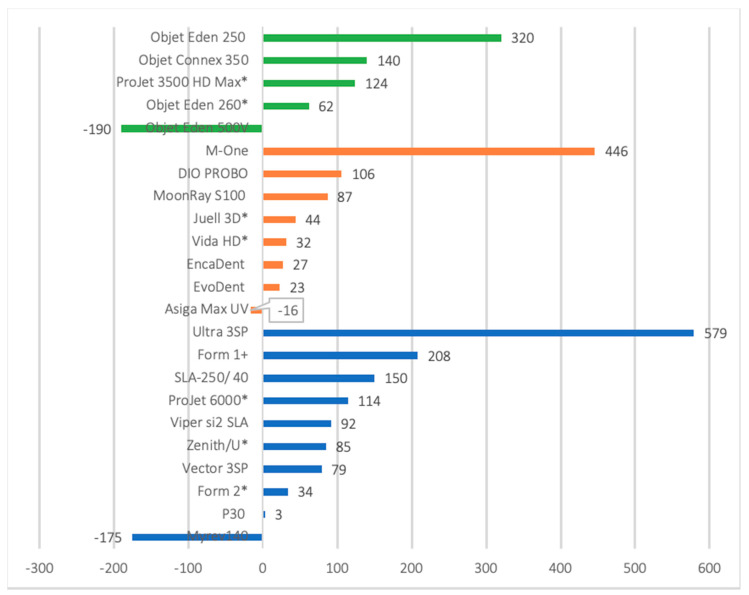
Reported trueness in microns for material jetting (MJ, green), digital light processing (DLP, orange) and stereolithography (SLA, blue) 3D printed full-arch dental models. * Asterisk denotes lowest mean error identified from different studies—other results in microns reported include: Form 2 = 59, 64 and −80; Zenith series = 138; Projet 6000 = 190; Juell 3D = 44, 70; Vida = 56; Objet Eden 260 series = 74, 80 and 85; Projet 3500 HD Max = 129. Data from studies that did not report details of 3D printer used or trueness data were not included in the figure.

Overall, the mean deviations from the reference model across all studies ranged from 3.3 to 579 μm [7,39]. Studies which assessed the trueness of both 3D printed and stone models found that the mean error for the stone model was consistently lower than their 3D printed counterparts [4,6,34,35,40,45]. In contrast, one study [45] reported no statistical differences between stone and MJ models and another [6] found no statistical difference between SLA and stone. However, several studies did not fully report the details of the 3D printer/s used or their trueness results [38,40,41,44,46,49,55]. Nonetheless, six DLP printers, five SLA printers and one MJ printer had an error measurement of <100µm for full-arch dental models, demonstrating high trueness (Figure 2). Similarly, the BJ printer (ZPrinter 450, 3D Systems, USA), CLIP printer (M2, Carbon, USA) and two FFF printers (Ultimaker 2+, Ultimaker B.V, Geldermalsen, The Netherlands; and M2, Makergear, USA) reported high trueness results (Table 2).

All SLA printers consistently produced oversized 3D printed models compared to the control, excluding the Myrev 140 printer [50]. The P30 reported the lowest mean error of 3.3 μm [7] and the Form 2 printer followed with reported mean errors ranging between 34.4 to 64 μm [37,43,56]. The SLA Ultra 3SP demonstrated the highest mean error at 579 μm [39]. Similar results were found for DLP printers with the majority of printers producing oversized models, except the Asiga Max UV, which also reported the lowest mean error for DLP at—16 μm [50]. The Evodent was the second most accurate DLP printer with a 23.3 μm error, followed by the Encadent at 26.5 μm errors [50,57]. Furthermore, JUELL 3D FLASH OC, Vida HD and Vida had a reported mean error of 44 μm, 31.7 μm and 56μm, respectively [43,45,57]. The highest mean error for the DLP printing technology was the M-One printer with a mean error of 446 μm [19]. Within MJ printers, Objet Eden 260 series (V, VS) had the lowest mean errors ranging from 62 to 85 μm [19,36,42,43], whilst the highest mean error was 320 μm (Objet Eden 250) [54]. Ultimaker 2+ printer as FFF technology had the least deviation error of 12μm [50], while Cubicon 3DP 110F reported a mean error of 307 μm [19]. The two printers for BJ (Z printer 450 and unclear) and CLIP (Carbon M2) technologies had mean errors of—20 μm [56] and 48μm [53], respectively.

#### 3.4.3. Precision

The precision of 3D printed models was assessed in 10 studies using either root mean square value (RMS) [4,6,19,40,42,45], the intraclass correlation coefficient (ICC) [38,53] and or interquartile range (IQR) [50]. The RMS value ranges for SLA, FFF, MJ and DLP were 23 to 91 μm, 52.1 to 99 μm, 38 to 68 μm and 53.8 to 76 μm, respectively. The range for ICC was 0.968 for CLIP and 0.999 for MJ. In addition, one study [50] reported IQR of 28 to 134 μm for SLA, 55 to 56 μm for FFF and 47 μm for DLP.

Two studies found 3D printed models to have equal or greater precision than conventional stone models [6,45]. By contrast, two studies [4,40] found conventional stone models to be more precise than the 3D printed models. Of note, studies that used ICC [38,53] to assess precision; demonstrated excellent reproducibility (>0.9 ICC value) of 3D printed models, according to the Koo and Li (2016) classification [58].

### 3.5. Statistical Analysis

The limited reporting, varying printing technologies, printing parameters, assessment methodology and statistical analysis employed in the included studies presented a heterogeneity that precluded from performing a meaningful meta-analysis.

### 3.6. Risk of Bias Assessment

The risk of bias and applicability concerns varied across the studies, which may have influenced the reliability of their results (Table 3). The reference standards used in almost all the studies had a low risk of bias and low concerns regarding applicability (27/28). The risk of the index test, however, was high for the majority of studies (21/28). This high risk was because the studies either did not use 3D superimposition, and therefore the mean error may not have been an accurate representation of the whole arch deviation, or the method of assessing the model’s deviation introduced errors other than those arising from the CAM process. These errors include the use of full-arch intraoral scanning for data acquisition which may introduce scanner error in-addition to the 3D printing error. Similarly, lack of details of assessors and their calibration was a noted risk of bias in several studies. Finally, the majority of studies had a high risk of bias for sample selection. This high risk is attributed to the lack of details relating to sample size calculation, spectrum of selected samples and/or postprocessing protocol. However, most of the samples remained highly applicable with the measurement protocol employed in the studies appropriately described to allow the reviewer to answer the review question.

## 4. Discussion

Given 3D printing’s promising potential and increased use in dentistry, it is essential to evaluate the accuracy of 3D printed dental models. This is the first systematic review, to the authors’ knowledge, investigating the accuracy of dental models manufactured using 3D printing technology. The selection criteria for the included reference standards were high, subsequently the risk of bias and applicability concerns were low according to the QUADAS-2 tool. The findings of this review support the use of 3D printing for the fabrication of dental models and deem them as clinically acceptable with the majority of included studies (*n* = 20/28) establishing a clinically acceptable error range of <100 to 500 μm. 3D printed models were found to be a valid alternative to stone models when taking precision into account. Nonetheless, the study by Wan Hassan (2019) was an outlier which found BJ 3D printed models not clinically acceptable due to their discrepancy of >500 μm. It is, however, worth noting the included studies which used orthodontic models [19,34,36,42,46,47,49,50,52,55,57] had more relaxed thresholds for clinical acceptability (up to 500 μm), compared to those intended for prosthodontic applications (up to 200 μm) [6,51,53]. Indeed, in orthodontics, a measurement difference of <300 µm between orthodontic casts and 3D printed models has been reported to be clinically acceptable [59,60,61]. On the other hand, in prosthodontics, the accuracy needs of dental models for the fabrication of dental prostheses is generally considered higher. A recent study concluded that three-unit fixed partial dentures fabricated using 3D printed models, whilst demonstrating inferior fit when compared to those fabricated using stone casts [27], the detected marginal gaps remained within the clinically accepted threshold of 120 µm reported in the literature [62]. Such clinically relevant thresholds become more critical in complex prosthodontic treatment modalities. Implant-supported complete dental prostheses or hybrid bridges have a maximum acceptable threshold of fit between the prostheses platform and the dental implants ranging between 59–150 µm [63,64,65]. Accordingly, the choice of 3D printing technology must be determined by its intended application. Hence, it is reasonable to conclude that 3D printed models which are clinically acceptable for orthodontic purposes may not necessarily be acceptable for the prosthodontic workflow or other dental applications requiring high accuracy.

The most common 3D printing technology investigated by the included studies was SLA with the findings demonstrating that SLA and DLP achieved the best accuracy for full-arch models. Amongst the SLA printers, Form 2 by Formlabs was investigated the most, and consistently produced clinically acceptable models. Although a wider range of mean errors was observed amongst SLA printed models, the Form 2 SLA desktop printer [43,49,51,57] also consistently produced models more accurate than MJ printers and was more cost-effective [43,44]. Moreover, the SLA printer P30 reported the most accurate models amongst all studies, followed by the DLP Asiga Max UV [7,50]. Additionally, SLA printers produced acceptable results regardless of their layer thickness, and therefore the layer thickness of 100 μm may be considered as an optimal thickness that balances accuracy and printing time when compared to 25 and 50 µm layers [49,57]. Moreover, it was suggested that a hollow or honeycomb infill could be indicated to reduce printing time and material-use with study models. Although no studies assessed the effect of using different resins with the same printer, using the manufacturer recommended resin was advised. In contrast, only one study assessed CLIP technology and used the Carbon M2 printer, which printed 3D models with deviations as small as 48 μm [53]. This study also concluded that the accuracy of 3D printed models was affected by the printing technique regardless of the base design. However, due to the limited studies that assessed the accuracy of BJ [56] and CLIP technologies [53], further investigation of these techniques is required to validate the viability of these printers. It is worth mentioning that some studies did not provide details of the sample size calculation, resin materials and/or post-curing protocols (Table 3), exposing them to high risk of bias and applicability concerns with regards to sample selection. As a result, no conclusions were drawn based on these parameters, other than those studies that reported using the manufacturer’s recommendations.

The two studies which examined the Ultra printer by EnvisionTEC [38,39] reported that the SLA models with horseshoe bases were not accurate nor clinically acceptable due to contraction in the transversal dimension during the post-curing protocol. However, as the horseshoe base is favoured for appliance fabrication and reduces material use, the inclusion of a posterior connection bar was suggested to prevent this significant dimensional reduction in the posterior region of the SLA model [37,38]. Nevertheless, several studies assessing other SLA printers [4,34,37,41,43,50,51] contradicted these findings and concluded that models printed by SLA with a horseshoe base to be clinically acceptable.

When assessing DLP technology, apart from the M-One printer used by Kim et al. (2018), all other printers had accuracies comparable to SLA and MJ. The Asiga Max UV printer produced the lowest mean error (−16 μm) [50]. In addition, Sherman et al. (2020) and Zhang et al. (2019) assessed the accuracy of DLP printed models with various layer thicknesses ranging from 20–100 μm and suggested that all the printed models were clinically acceptable. Thus, similar to SLA printers, it can be inferred that a layer thickness of 100 μm can still produce models with clinically acceptable accuracies for DLP printers. In addition to layer thicknesses, two studies assessed different filling patterns for DLP printed models [53,55]. Altering the filling pattern from solid to hollow reduced material wastage, build time and cost with no statistically significant difference in mean error.

Most MJ printers could reproduce models with high levels of trueness and precision, regardless of their base design [38]. From those, Objet Eden 260 series [19,36,42,43], was the most commonly investigated printer and consistently produced models with the highest accuracies due to its smaller layer thickness of 16 μm followed by the Projet3500 HDMax [6,45]. These printers were used due to their relatively affordable price and ability to print in smaller layer thicknesses. It is worth mentioning that although the reduction in layer height resulted in smoother surface finish and greater detail, the printing time increased [43].

FFF desktop printers, albeit considered the most affordable printers [46,50], provided models with acceptable accuracy. The most accurate models were created by the Ultimaker 2+ printer (12 μm) [50]. Although the materials used by FFF printers, namely PLA or ABS were inexpensive; the resultant models had inferior surface properties compared to acrylates and epoxides which were used for vat polymerisation technologies (SLA, DLP and CLIP). Similar to SLA and DLP, studies assessing FFF suggested a layer thickness of 100 μm to be clinically acceptable. Moreover, Burde et al. (2017) printed FFF models with a honeycomb pattern to reduce print time, material and cost with the resultant models deemed clinically acceptable.

There were very limited data to compare the results from 3D assessment to linear measurements for the same printers. However, it is worth noting that the highest risk of bias and applicability concerns for index test were recorded for studies that used linear measurements. This was reflective of the limited measuring points provided by those studies in comparison to a full arch deviation measurement by 3D superimposition. Additionally, some of the studies had a high risk of bias as human error may have been introduced by performing physical linear measurements with no information provided on the calibration of the examiners [19,49,50,53]. Furthermore, for 3D superimposition techniques, the risk of bias and applicability concerns were low for most studies as high accuracy desktop scanners were utilised and CAM was the only identified source of error. Nevertheless, studies that used intraoral scanners, made conventional impressions with or without pouring casts had a higher risk of bias due to the additional stages that may have introduced their own set of errors.

The Projet 6000 printed models were assessed using different methods [6,34]. The mean error calculated using full arch 3D superimposition (114.3 μm) was smaller than the intermolar width error measured by a surveying software (190 μm). Similarly, two studies assessed the Juell 3D printer [36,43], and the mean error calculated by full arch superimposition (44 μm) was smaller than the digital calliper measurements for the intermolar width (70 μm). On the other hand, two studies [19,36] assessed the Objet Eden 260VS model, using two different linear measurement methods. The mean errors calculated using surveying software and digital calliper were very similar (74 and 80 μm, respectively). These findings do highlight the need for a standardised measuring protocol to facilitate comparison of results across studies given the noted discrepancy between the different assessment techniques.

A potential limitation of this review is the assessment findings of the included studies in relevance to the measurement time of the 3D printed models. This limitation is due to the possible dimensional changes exhibited by printed models over time, with only six of the included studies identifying the time of model measurement. Joda et al. (2020) [7] assessed the effect of time on the accuracy of the printed models and was the solely identified study that reported assessing the models for more than one week. The results suggested that the accuracy of SLA printed models was time-dependent due to a statistically significant change in their dimensions after three weeks of storage, suggesting the use of SLA 3D printed models as single-use products with definitive prosthetic reconstructions. The lack of standardised reporting in included studies is also a limitation that may have resulted in a high risk of bias in terms of index test and sample selection.

Consequently, the evident heterogeneity of the included studies with varying techniques, manufacturing parameters, materials and assessment protocols, a meta-analysis was not feasible. It is also worth noting the limitations present in the literature which need to be addressed in future studies. Investigation of different layer thicknesses for FFF, MJ, BJ and CLIP printing technologies, the effect of time and storage conditions on the accuracy of different 3D printed models, as well as clinical patient outcomes, remain lacking. A standardised accuracy assessment protocol for 3D printing of dental models is also necessary to facilitate performance comparison. Future studies should also involve a standardised reporting protocol that details all printing parameters, materials used, postprocessing protocol and time of assessment.

## 5. Conclusions

The findings of this study support the use of 3D printed dental models, especially as orthodontic study models. Irrespective of the 3D printing technology, certain printers were able to demonstrate low errors and hence can be recommended for dental applications that require high accuracy models. Other factors such as layer thickness, base design, postprocessing and storage can equally influence the accuracy of the resultant 3D printed models. Nonetheless, the high risk of bias with regards to the lack of standardised testing of accuracy warrants careful interpretation of the findings.

## Figures and Tables

**Table 1:** Search strategy.

1. Search (print * OR “rapid prototyping” OR “additive manufacturing” OR fabrication OR stereolithography OR “stereo-lithography” OR “stereo lithography” OR photopolymer * OR photopolymer * OR “fused deposition Ωmodelling” OR “fused filament fabrication” OR “material extrusion” OR “material jetting” OR photojet OR polyjet OR “photopolymer jetting” OR “multijet printing” OR “binder jetting” OR “digital light processing” OR “selective laser sintering” OR “continuous liquid interface production” OR photopolymer * OR RP OR AM OR SLA OR SL OR FDM OR FFF OR PPJ OR PJ OR MJP OR MJ OR DLP OR CLIP OR SLS)
2. Search (“dental cast *” OR “dental model *” OR edentulous * OR edentate * OR dentate OR “full arch” OR “replica cast *”) AND (3 D OR 3D OR 3 dimensional OR three dimensional)
3. Search (accuracy OR accuracies OR applicability OR precision OR repeatability OR reproducibility OR trueness OR sensitivity OR specificity OR specificities OR validation OR validity OR value OR agreement OR “spatial error *” OR “geometric error *” OR “dimensional error *” OR correctness OR exactness)
4. Search ((#1 and #2 and #3)) Filters: Publication date from 01/01/2005 to 13/05/2020

**Table 2:** Details of studies included in the systematic review.

Authors/Date	3D printing Material	Model Data Source	Model Data Source	3D Printing System	3D Printer Details	Resolution x, y, z (µm)	Sample Size (Single Arch/Printer)	Assessment Method	Trueness (SD) (µm)	Precision (µm, ICC and IQR)
Aly and Mohsen, 2020 [34]	Photocurable polymer (liquid resin)	IOS scanned full dentate Typodont (Mx and Md)	IOS scanned Typodont	SLA	ProJet 6000, 3D Systems	Unclear	10	Digital callipers Tooth: MD, CH Arch: IC, IM	190 (100)	Unclear
Bohner et al. 2019 [35]	Unclear	Typodont (Mx, 7–7) containing implants at sites of 21, 24 and 26	Typodont (maxillary)	SLA	Unclear, Envisiontec	Unclear	10	Surveying software Arch: IP, IM	19.7 (13.3)	Unclear
Brown, Currier, Kadioglu and Kierl, (2018) [36]	Unclear	Patient IOS and alginate impressions (Mx and Md, min 6–6) 30 cases	Patient IOS and alginate impressions 30 cases	DLP MJ	Juell 3D Flash OC, Park Dental Research Objet Eden 260VS, Stratasys	z: 50, 100 z: 16	60	Digital callipers Arch: IC, IM, AD Tooth: MD, CH Occlusion: Unclear	70 80	Unclear
Burde et al. (2017) [37]	Poly-L-lactic acid wire Poly-L-lactic acid wire Grey light-curing resin	Patient stone model (Mx and Md) 10 cases. Unclear number of teeth present	Patient stone model 10 cases	FFF FFF SLA	Creatr HS, Leapfrog Custom RepRap, (based on a PrusaI3 kit) Form 1+, Formlabs	z: 100 z: 100 z: 25	20	3D assessment Nominal ±11.51 Critical: ±230	156.2 (22.4) 128.3 (18.3) 207.9 (44.6)	Unclear
Camardella, de Vasconcellos Vilella and Breuning, (2017) [38]	Photopolymer resin Light-curing methacrylic resin (E-Denstone; Envisiontec)	Patient IOS 10 cases (Mx and Md, min 7–7)	Patient IOS 10 cases (mandibular)	MJ SLA	Objet Eden 260VS, Stratasys Ultra 3SP Ortho, Envisiontec	z: 16 z: 100	20	Surveying software Arch: IC, IP, IM Tooth: Unclear Occlusion: Unclear	Unclear	0.999ICC 0.998 ICC
Camardella, Vilella, van Hezel and Breuning, (2017) [39]	Light curing methacrylic resin (RC31, Envi- siontec)	Patients IOS and impressions (Mx and Md, min 6–6) 28 cases	Patients IOS and impressions 28 cases	SLA	Ultra 3SP, Envisiontec	Unclear	56	Digital callipers Arch: IC, IM Tooth: MD, CH Occlusion: OJ, OB AND 3D assessment Nominal: ±50 Critical: ±500	579 (1050)	Unclear
Cho, Schaefer, Thompson and Guentsch, (2015) [40]	Unclear	Lab scanned fully dentate Typodont (Mx) with 5 prepared teeth (16, 15, 21, 23, 26)	Lab scanned Typodont (maxillary)	SLA	Unclear	Unclear	5	3D assessment Nominal: ±50 Critical: ±500	27 (7)	91 (10)
Choi, Ahn, Son and Huh, (2019) [4]	Photopolymer Photopolymer	Typodont (Mx, 7–7) with prepared teeth (16, 11, 24 and 26)	Typodont (maxillary)	SLA DLP	ZENITH U, Dentis DIOPROBO, DIO	z: 50 z: 50	10	3D assessment Nominal: ±50 Critical: ±500	85.2 (13.1) 105.5 (22.5)	49.6 (12.1) 53.8 (17.5)
Cuperus et al. (2012) [41]	Epoxy Resin	IOS Dry human skull (min 6–6, with max 1 missing or deciduous tooth per skull) 10 cases Intra-oral scanner	IOS Dry human skull 10 cases	SLA	Unclear	Unclear	20	Digital callipers Arch: IC, IM Tooth: MD Occlusion: Unclear	100	Unclear
Dietrich, Ender, Baumgartnerand Mehl, (2017) [42]	Epoxy-based resin (Accura) photopolymer resins	Patient IOS 2 cases (Mx). Unclear number of teeth present	Patient IOS 2 cases (maxillary)	SLA MJ	Viper si2 SLA, 3D Systems Objet Eden 260, Stratasys	z: 100 at base and 50 at tooth level z: 16	10	3D assessment Nominal: ±20 Critical: ±100	92 (23) 62 (8)	20 (4) 38 (14)
Favero et al. (2017) [43]	Grey photopolymer resin (FLGPGR02; Formlabs). Unclear	Typodont (Mx, 7-7)	Typodont (maxillary)	SLA SLA DLP DLP MJ	Form 2, Formlabs Vector 3sp, Envisiontec Juell 3D, Park Dental Perfactory Desktop Vida, Envisiontec Objet Eden 260V, Stratasys	z: 25, 50, 100 z: 100 z: 100 z: 100 z: 28	12	3D assessment Nominal: ±20 Critical: ±250	64 79 44 56 85	Unclear
Hazeveld, Huddleston Slater and Ren, (2014) [44]	Unclear	Patient Stone model (Mx and Md, min 6–6) 6 cases	Patient Stone model 6 cases	DLP BJ MJ	Unclear, Envisiontec, Unclear, Z-Corp Unclear, Objet Geometries	Unclear	12	Digital callipers Arch: Unclear Tooth: MD, CH Occlusion: Unclear	Unclear	Unclear
Jin, Jeong, Kim and Kim, (2018) [45]	Unclear	Lab scanned Typodont (Mx, 7–7)	Lab scanned Typodont (maxillary)	MJ FFF	ProJet 3500 HDMax, 3D Systems Cube, 3D Systems	z: 31.97 z: 123.71 (thickness measured after printing)	10	3D assessment Nominal: ±50 Critical: ±500	129.1 (7.8) 149.0 (4.7)	44.6 (8.9) 52.1 (10.9)
Jin, Kim, Kim and Kim, (2019) [6]	Photocurable liquid resin Acrylic polymer	Lab scanned Typodont (Mx and Md, 7–7)	Lab scanned Typodont (maxillary)	SLA MJ	ProJet 6000, 3D Systems ProJet 3500 HD Max, 3D Systems	FMR FMR	10	3D assessment Nominal: ±50 Critical: ±500	114.3 (1.8) 124 (3.7)	59.6 (8.2) 41.0 (5.8)
Joda, Matthisson and Zitzmann, (2020) [7]	Light-curing polymer, (SHERAPrint-model plus “sand” UV, SHERA)	IOS Typodont (Mx, 7–7), with missing 25 and prepared 24 and 26)	IOS Typodont (maxillary)	SLA	P30, Straumann	Unclear	10	3D assessment Nominal: unclear Critical: unclear	3.3 (1.3)	Unclear
Kasparova et al. (2013) [46]	ABS plastic material, Clear resin	Patient stone model 10 cases. Unclear number of teeth present	Patient stone model 10 cases	FFF MJ	RepRap, Unclear ProJetHD3000, 3D Systems	x,y: 200, z: 0.35 Unclear	20 2	Digital callipers Tooth: CH Arch: IC	Unclear Unclear	Unclear Unclear
Keating, Knox, Bibb and Zhurov, (2008) [47]	Hybrid epoxy-based resin	Patient stone model 15 cases. Unclear number of teeth present	Patient stone model 15 cases	SLA	SLA-250/40, 3D Systems	z: 150	30	Digital callipers Tooth: CH Arch: IC, IP, IM	150 (160)	Unclear
Kim et al. (2018) [19]	Unclear	Lab scanned Typodont (Mx and Md, 7–7)	Lab scanned Typodont	SLA: DLP MJ FFF	ZENITH, Dentis M-One, MAKEX Technology Objet Eden 260VS, Stratasys Cubicon 3DP-110F, HyVISION System	x,y: 50 z: 50 x,y: 70 z: 75 z: 16 x,y: 100 z: 100	10	Surveying software Tooth: MD, BL, CH Arch: IC, IM	138 (79) 446 (46) 74 (39) 307 (61)	88 (14) 76 (14) 68 (9) 99 (14)
Kuo, Chen, Wong, Lu and Huang, (2015) [48]	Unclear	Patient IOS Patient impressions poured, and lab scanned (Md, 7–7) 1 case	Patient IOS Patient impressions poured, and lab scanned 1 case	MJ	Connex 350, Stratasys	Unclear	1	3D assessment Nominal: ±60 Critical: ±300	140	Unclear
Loflin et al. (2019) [49]	Grey photopolymer resin, (FLGPGR03; Formlabs)	Patient stone models (Mx and Md) 12 cases. Unclear number of teeth present	Patient stone model 12 cases	SLA	Form 2, Formlabs	z: 25, 50,100	24	ABO tool Tooth: marginal ridge Occlusion: OJ, occlusal contacts	Unclear	Unclear
Nestler, Wesemann, Spies, Beuer and Bumann, (2020) [50]	Dental SG Optiprint Imprimo LC model ABS Polylactide	Cast in standard tessellation language (STL) format (Mx, 7–7) including 5 measuring cubes in areas 16, 26, 13, 23 and between 11 and 21)	Maxillary cast in standard tessellation language (STL) format	SLA SLA DLP FFF FFF	Forms 2, Formlabs Myrev140, Sisma Asiga Max UV, Asiga M2, Makergear Ultimaker 2+, Ultimaker	Unclear Unclear Xy: 62, Z: Unclear Unclear x,y: 12.5, z: Unclear	37 34 for Myrev140	Surveying software Arch: IC, IM, arch length	-80 (94 −175 (28) −16 (32) −55 (39) 12 (43)	134 28 47 55 56
Papaspyridakos et al., (2020) [51]	Photopolymer resin, dental model resin (Formlabs)	Lab scanned Patient stone model 1 case (Md) with 4 abutment-level implant analogs	Lab scanned Patient stone model 1 case (mandibular)	SLA	Form 2, Formlab	z: 25	25	3D assessment Nominal ±50 Critical: ±200	59 (16)	Unclear
Rebong, Stewart, Utreja and Ghoneima, (2018) [52]	Unclear	Patient stone models (Mx and Md, min 6–6) 12 cases	Patient stone model 12 cases	FFF SLA MJ	Makerbot Replicator, Makerbot Industries Projet 6000, 3DSystems Objet Eden 500V, Stratasys	z: 100 z: 50 z: 16	24	Digital calipers Arch: IC, IM Tooth: Unclear Occlusion: OJ, OB	110 (420) −20 (370) −190 (330)	Unclear
Rungrojwittayakul et al. (2020) [53]	Unclear	Lab scanned fully dentate Typodont (Mx,)	Lab scanned Typodont (maxillary	CLIP DLP	Carbon M2, Carbon MoonRay S100, SprintRay	Unclear	10	3D assessment Nominal: ±10 Critical: ±100	48 (44) 87 (57)	0.968 ICC 0.983 ICC
Saleh, Ariffin, Sherriff and Bister, (2015) [54]	Unclear	Lab scanned Typodont (Mx and Md, 7–7)	Lab scanned Typodont	MJ	Objet Eden 250, Stratasys	Unclear	8	Digital calipers Tooth: MD Arch: IC, IM Occlusion: OJ, OB	320 (156)	Unclear
Sherman, Kadioglu, Currier, Kierl and Li, (2020) [55]	Unclear	Patient IOS (Mx and Md, min 6–6) 15 cases	Patient IOS 15 cases	DLP	JUELL 3D Flash OC, Park Dental Research Corporation	z: 50, 100	30	Digital calipers Arch: IC, IM, AD Tooth: MD, CH Occlusion: Unclear	Unclear	Unclear
Wan Hassan, Yusoff and Mardi, 2017 [56]	High-performance composite (Zp151; 3D Systems).	Patient impression (Mx and Md, min 6–6) 10 cases	Patient impression 10 cases	BJ	Z Printer 450, 3D Systems	z: 89–102	30	Digital callipers Arch: IC, IP, IM Tooth: MD, CH, BL Occlusion: Unclear	−20	Unclear
Zhang, Li, Chu and Shen, (2019) [57]	Dental model resin (Formlabs) Model Ortho resin (Union Tec) Encashape, ENCA-Model resin Light curing methacrylate resin E-Denstone, EnvisionTEC	Patient IOS (Mx and Md, 7–7) 1 case	Patient IOS 1 case	SLA DLP DLP DLP	Form 2, Formlabs EvoDent, UnionTec EncaDent, Encashape Vida HD, EnvisionTec	x,y: 140 z:25, 30,10 z: 50,100 x,y: 58 z: 20, 30, 50,100 x,y: 50 z: 50, 100	2	3D assessment Nominal: ±50 Critical: ±250	34.4 23.3 26.5 31.7	Unclear

Mx = maxillary, Mn = mandinular, CH = crown height, BL = buccolingual width, MD = mesiodistal width, IC = intercanine width, IP = interpremolar width, IM = intermolar width, OB = overbite, OJ = overjet, SLA = stereolithography, MJ = material jetting, BJ = binder jetting, DLP = digital light processing, CLIP = continuous liquid interface production, FFF = fused filament fabrication, IOS = intraoral scanner, ABO = American Board of Orthodontics.

**Table 3:** Risk of bias and applicability concerns according to QUADAS-2 tool. Negative sign (–) denotes high risk of bias. Positive sign (+) denotes low risk of bias.

Study	Risk of Bias			Applicability Concerns		
	Patient (Sample) Selection	Index Test	Reference Standard	Patient (Sample) Selection	Index Test	Reference Standard
Aly and Mohsen, 2020 [34]	−	−	+	+	−	+
Bohner et al. 2019 [35]	−	−	+	−	−	+
Brown, Currier, Kadioglu and Kierl, 2018 [36]	−	−	+	+	−	+
Burde et al. 2017 [37]	−	+	+	+	+	+
Camardella, de Vasconcellos Vilella and Breuning, 2017 [38]	+	−	+	+	−	+
Camardella, Vilella, van Hezel and Breuning, (2017) [39]	−	−	+	+	−	+
Cho, Schaefer, Thompson and Guentsch, 2015 [40]	−	+	+	−	+	+
Choi, Ahn, Son and Huh, 2019 [4]	−	+	+	+	+	+
Cuperus et al. 2012 [41]	−	−	+	−	−	+
Dietrich, Ender, Baumgartner and Mehl, 2017 [42]	−	−	+	+	−	+
Favero et al. 2017 [43]	−	+	+	+	+	+
Hazeveld, Huddleston Slater and Ren, 2014 [44]	−	−	+	−	−	+
Jin, Jeong, Kim and Kim, 2018 [45]	−	+	+	+	+	+
Jin, Kim, Kim and Kim, 2019 [6]	−	+	+	+	+	+
Joda, Matthisson and Zitzmann, 2020 [7]	−	−	+	+	−	+
Kasparova et al. 2013 [46]	−	−	+	+	−	+
Keating, Knox, Bibb and Zhurov, 2008 [47]	−	−	+	+	−	+
Kim et al. 2018 [19]	−	+	+	+	+	+
Kuo, Chen, Wong, Lu and Huang, 2015 [48]	−	−	+	+	−	+
Loflin et al. 2019 [49]	−	−	+	+	−	+
Nestler, Wesemann, Spies, Beuer and Bumann, 2020 [50]	−	-	+	+	−	+
Papaspyridakos et al., 2020 [51]	+	−	+	+	−	+
Rebong, Stewart, Utreja and Ghoneima, 2018 [52]	−	−	+	+	−	+
Rungrojwittayakul et al. 2020 [53]	−	−	+	+	−	+
Saleh, Ariffin, Sherriff and Bister, 2015 [54]	−	−	+	+	−	+
Sherman, Kadioglu, Currier, Kierl and Li, 2020 [55]	−	−	+	+	−	+
Wan Hassan, Yusoff and Mardi, 2017 [56]	+	−	+	+	−	+
Zhang, Li, Chu and Shen, 2019 [57]	−	−	+	+	−	+
